# Pre-operative sera interleukin-6 in the diagnosis of high-grade serous ovarian cancer

**DOI:** 10.1038/s41598-020-59009-z

**Published:** 2020-02-10

**Authors:** Nirmala Chandralega Kampan, Mutsa Tatenda Madondo, John Reynolds, Julene Hallo, Orla M. McNally, Thomas W. Jobling, Andrew N. Stephens, Michael A. Quinn, Magdalena Plebanski

**Affiliations:** 1 Department of Immunology & Pathology, Monash University, Central Clinical School, Level 6, 89, Commercial Road, Melbourne, 3181 VIC Australia; 2 Gynae-oncology Unit, Royal Women’s Hospital, 20 Flemington Road, Parkville, Melbourne, Victoria 3052 Australia; 3 Department of Obstetrics and Gynaecology, Universiti Kebangsaan, Malaysia Medical Centre, Kuala Lumpur, Malaysia; 4 Biostatistics Consulting Platform, Central Clinical School, Monash University, Central Clinical School, Level 6, Commercial Road, Melbourne, 3181 VIC Australia; 5 Department of Obstetrics and Gynaecology, University of Melbourne, Victoria, 3052 Australia; 6 Epworth Research Institute, Epworth Healthcare, Richmond, 3121 Australia; 7 Centre for Cancer Research, Hudson Institute of Medical Research, 27-31 Wright St, Clayton, VIC 3168 Australia; 8 Department of Molecular and Translational Sciences, Monash University, Clayton, 3168 Australia; 9 School of Health and Biomedical Sciences, RMIT University, Melbourne, Australia

**Keywords:** Tumour biomarkers, Interleukins, Diagnostic markers, Tumour biomarkers

## Abstract

Pre-operative discrimination of malignant masses is crucial for accurate diagnosis and prompt referral to a gynae oncology centre for optimal surgical intervention. HGSOC progression is correlated with local and systemic inflammation. We hypothesised that inclusion of inflammatory biomarkers in sera may improve diagnostic tests. In the training cohort, we tested four existing clinical tests (RMI score and ROMA, CA125 and HE4) and a panel of 28 immune soluble biomarkers in sera from 66 patients undergoing surgery for suspected ovarian cancer. Six promising immune biomarkers alone, or in combination with conventional tests, were subsequently analysed in an independent validation cohort (n = 69). IL-6 was identified as the main driver of variability followed closely by conventional diagnostic tests. Median sera IL-6 was higher in HGSOC patients compared to those with a benign mass or controls with normal ovaries (28.3 vs 7.3 vs 1.2 pg/ml, p < 0.0001). The combination of IL-6 further improved the overall predictive probability of the conventional tests. Modelling a two-step triage of women with a suspicious ovarian mass, with IL-6 > 3.75 pg/ml as primary triage followed by conventional tests (CA125 or RMI score) identified ovarian cancer in patients with a misclassification rate of 4.54–3.03%, superior to the use of CA125 or RMI alone (9.09 to 10.60). The validation cohort demonstrated a similar improvement in the diagnostic sensitivity following addition of IL-6. IL-6 in combination with conventional tests may be a useful clinical biomarker for triage of patients with a suspected malignant ovarian mass.

## Introduction

Epithelial ovarian cancer is one of the leading causes of death globally. High-grade serous ovarian carcinoma (HGSOC) is the most aggressive subtype and generally presents at an advanced stage^1^. Although the 5-year survival rate increased significantly from 36% in 1986–1990 to 46% in 2011–2015^2^, the prognosis for women with advanced ovarian cancer remains poor due to its late presentation and the lack of scientifically validated screening tools^2,3^. Optimal surgical cytoreduction as part of a multidisciplinary team, remains an important prognostic factor in ovarian cancer management and survival^4^. Pre-operative discrimination of ovarian carcinoma from non-malignant pelvic masses is crucial to ensure correct diagnosis and prompt referral to a specialist center, and subsequent optimization of primary treatment to improve survival.

A definitive predictive biomarker has not yet been identified. Standard investigation tools for adnexal masses currently in place are clinical examination, assays of tumour markers and ultrasound assessment. None of these tools is very sensitive or specific for predicting malignancy when considered separately.

Serum CA125, a glycoprotein antigen, is found at elevated levels in 1% of healthy individuals, 6% of patients with benign ovarian mass, as well as other malignancies^5^ and physiological states, including pregnancy, endometriosis and menstruation^6^. Serum CA125 levels are raised above 35 U/mL in 78% of women with malignant ovarian mass, but also in 22% of women with benign masses^7^. Therefore, serum CA125, when used alone, is not highly reliable in differentiating patients with malignant compared to benign masses^8^. In a review by Jacobs and Bast *et. al*, while only about 50% of patients with stage I epithelial ovarian cancer had elevated levels of CA125, highest levels were seen in more than 90% of women with advanced stages^6^. CA-125 has greater sensitivity and specificity in postmenopausal women than in those who are premenopausal. In a study by Grzybowski W *et. al*, the sensitivity and specificity of CA125 among postmenopausal women with ovarian cancer were higher at 88.7% and 98.07% respectively, while in the premenopausal cohort sensitivity was only 64.0% and specificity 94.1%^9^.

The Risk of Malignancy Index (RMI), an algorithm developed by Jacobs *et al*. in 1990^10^, is the most widely used risk assessment for ovarian malignancy and is valuable for selective referral to a specialist center. RMI (at a threshold of >200) has an improved sensitivity of 87% and specificity of 97% for ovarian cancer^11^ compared to CA125 alone. The RMI score is generated by a simplified regression equation of serum CA125 level, menopausal status scores and ultrasound feature scores (RMI = ultrasound findings x menopause status x CA125 U/ml)^10^. RMI stratifies patients into high or low risk based on a numerical score, with RMI >200 considered as high risk, with a likelihood of having a malignant tumour of 75%^12^, therefore requiring urgent assessment by a gynaecological oncologist to ensure optimal treatment outcome. Although the RMI score is highly sensitive and specific, it requires ultrasound imaging scoring^13^. In facilities with no ultrasound readily available, RMI scoring is underutilized to predict malignancy pre-operatively, and in facilities with multiple imaging modalities, the lack of standardization across imaging methods, and dependence on subjective operator assessment can highly influence the variability in RMI scores^13^.

Human epididymis protein 4 (HE4) is a relatively new serum biomarker for the detection of ovarian cancer^14,15^. HE4, a transcript of WFDC2 gene on chromosome 20^16^, was originally found to be expressed in human epididymis. HE4 is also expressed in normal tissues including respiratory and reproductive tract^17^. The overexpression of HE4 gene in ovarian cancer was discovered in 1999 by Shummer *et al*.^18^. In 2003, HE4 was reported as a promising serum biomarker for detection of ovarian cancer^19^. A meta-analysis of nine studies involving 1807 women reported that the pooled sensitivity and specificity for HE4 in diagnosing ovarian cancer were 83% and 90% respectively with a summarized ROC curve of 0.9271^20^. HE4 alone performed similarly when the ROC curve was compared with CA125, except CA125 performed better among postmenopausal women, while HE4 correlated inversely with age^15,21^. HE4 emerged as a potential lead biomarker out of nine analytes tested by Moore *et al*.^21^, who proposed it to be incorporated with CA125 and menopausal status into an algorithm to predict ovarian cancer. An alternative risk of malignancy algorithm (ROMA) combines CA125 and HE4 values along with the menopausal status into a predictive index, which in turn is used to calculate the predicted probability of ovarian cancer (from 0 to 100%). This has been shown to successfully stratify patients into high and low risk groups with 93.8% of ovarian cancer correctly classified as high risk^22^. The disadvantage of this algorithm is the use of HE4 which is an expensive test and is not readily available in low- or middle-income countries.

Components of the inflammatory and immunosuppressive pathway including systemic cytokines (interleukin (IL)-6, TNF-a, IL-10, TGF-*β*) and chemokines (CCL2, CCL4, CXCL10) in the ovarian cancer microenvironment have been shown to contribute to the development of carcinogenesis^23–25^. Cytokines and chemokines are cell signaling molecules that are essential to help cell to cell communication in the regulation of immunity, inflammation and hematopoiesis^23^.

We hypothesized that inclusion of inflammatory or immunosuppressive biomarkers present in serum or plasma may enhance or complement the identification of malignancy in patients with HGSOC, above current diagnostic methods including CA125, RMI, HE4 and ROMA.

## Material and Methods

### Trial design and patient details

#### Training phase

This study is a part of the Immunity and Ovarian Cancer trial (Project 13/32) and has been approved by the Human Research Ethics Committee (HREC) of the Royal Women’s Hospital, Melbourne. The clinical research study was carried out in accordance with relevant guidelines and regulations. A total of 80 women undergoing ovarian removal surgery and who fulfilled the study inclusion criteria (Table 1) were initially recruited following signed written informed consent. Following the strict exclusion study criteria (Table 1), fourteen women were excluded from the study. The reasons for exclusion were significant concomitant heart, liver and vascular disease (n = 3), taking immunosuppressive drugs (n = 4) and having a concomitant active cancer such as breast and colorectal cancer (n = 3) as well as an ovarian cancer other than high-grade serous type (n = 4). Sixty-six women were finally enrolled and constituted the final sample for the training phase of the study. Thirty-three women had newly diagnosed HGSOC, 12 women had benign ovarian masses and the control group consisted of 21 women undergoing risk reduction surgery for a known genetic mutation (e.g. BRCA or Lynch syndrome) or a strong family history of ovarian and/or breast cancers.

**Table 1:** Study criteria.

Inclusion criteria	Exclusion criteria
Age 18-80	Age <18 or >80 years
Signed written informed consent	Unable to give informed consent
Newly diagnosed, Stage III-IV, high-grade serous ovarian cancer (HGSOC) or benign ovarian tumour or normal ovaries	Pregnant
	Cancer other than Stage III-IV HGSOC
	Concurrent other active cancers
	Concurrent significant pre-existing major medical conditions (such as heart, liver or vascular diseases)
No prior chemotherapy or radiotherapy	Major surgery, open biopsy or significant trauma or injury within 28 days prior to sampling
	Receiving NSAIDS, anti-inflammatory steroids or immunosuppressant agents within 14 days prior to sampling
	Active inflammation, significant trauma or open wound

All relevant clinical information including age, self-reported menopausal status, pre-existing medical conditions and drug history, and any prior history of malignancy were obtained from de-identified patient medical records. Venous blood samples were obtained from patients prior to any surgical or chemotherapy treatment. Baseline blood components, serum CA125 levels and pelvic ultrasound reports were collected for all patients. Following surgery, relevant documentation on final diagnosis, surgical staging findings and a thorough histological assessment of tumour type, stage and grade following multidisciplinary team consensus were obtained. Patient’s tumours were staged according to the criteria of the International Federation of Gynecology and Obstetrics (FIGO). Patient details immediately relevant to this study are provided in Table 2.

**Table 2:** Characteristics of primary cohort patients (N = 66).

	Ovarian cancer n = 33	Benign ovaries n = 12	Normal ovaries n = 21	p value	Post-hoc Adjusted p value
**Demographic data**					
Age (years)					
Mean ± SD	60.1 ± 9.1	54.8 ± 10.6	51 ± 10.1	0.001	^a^p = 0.30
Median	60.0	55.0	48.0		^b^p = 0.001
Range	41–83	38–73	40–84		^c^p = 0.66
Body Mass Index (BMI) (kg/m^2^)					
Mean ± SD	29.6 ± 4.8	31.3 ± 3.9	31.3 ± 6.2	0.42	
Median	28.0	32.0	30		
BMI range	24–40	24–36	24–43		
**Family history of breast/ovarian/hereditary cancer mutation**					
Yes	6	2	9	0.13	
No	18	4	7		
Unknown	9	6	5		
**Gene mutation**					
Not tested	16	8	7	0.18	
Tested	17	4	14		
BRCA 1 mutation	12	2	10	0.21	
BRCA 2 mutation	4	1	5	0.39	
Lynch syndrome	1	0	2	0.38	
**Medical comorbidities**					
*(one individual may have more than one disease)*	12	3	3	0.47	
• Hypertension	6	1	1	0.87	
• Type 2 diabetes	4	2	0	0.80	
• Hypercholesterolemia	3	1	0	0.80	
• Thyroid disorders (euthyroid)	1	0	0	0.73	
• Bronchial asthma (controlled)	5	0	0	0.73	
• Mild chronic back pain	3	1	0	0.80	
• History of treated breast, cervical and/or endometrial cancer 5–30 years ago	4	0	0	0.73	
**Past history of anxiety and depression not on medication**					
**Ovarian tissue histology**					
No pathology			21		
High-grade papillary serous adenocarcinoma	33				
**Benign**					
Serous cystadenoma		9			
Fibrothecoma		3			
**Stage of disease**					
**Ovarian cancer**					
IIIA	1				
IIIB	2				
IIIC	28				
IV	2				
**Laboratory parameters**					
Haemoglobin (Hb) (g/L)					
Mean ± SD	119.8 ± 16.2	121.3 ± 20.8	123.8 ± 15.7	0.74	
Median	122	121	122		
Range	80–147	90–170	93–158		
Platelet (x10^3^/μL)					
Mean ± SD	327.8 ± 139.6	261 ± 73.0	267 ± 58.7	0.22	
Median	296	229	263		
Range	101–654	173–413	172–369		
**White cell count (WCC) (x10**^**9**^**/L)**					
Mean ± SD	8.51 ± 2.8	9.31 ± 3.2	10.16 ± 3.3	0.15	
Median	8.5	10.2	10.2		
Range	3.7–16.2	4.4–16.4	5–17.7		
**Absolute Neutrophil Count (ANC)**					
(x10^9^/L)	5.57 ± 2.7	5.93 ± 2.9	6.86 ± 3.4	0.27	
Mean ± SD	4.5	5.49	6.28		
Median	2.1–13.3	2.7–13.1	2.3–14.1		
Range					
**Total Lymphocytes (x10**^**9**^**/L)**					
Mean ± SD	1.90 ± 0.60	2.72 ± 0.89	2.43 ± 0.65	0.001	^a^p = 0.005
Median	1.8	2.58	2.26		^b^p = 0.02
Range	1.0–3.0	1.7–4.5	1.6–4.0		^c^p = 0.99

±SD- Standard deviation, ^a^cancer vs benign, ^b^cancer vs normal, ^c^benign vs normal.

#### Validation phase

The validation phase consisted of clinical samples obtained through the OCRF-sponsored Ovarian Cancer Tissue Banking program located at the Hudson Institute, Australia. Ethical approval was obtained from the Epworth Human Research Ethics Committee EH2016-165. The clinical research study was carried out in accordance with relevant guidelines and regulations. All participants provided prior informed written consent. A total of 50 women undergoing surgery for a suspected ovarian mass during the period 2014–2016 were included in the study. For independent sub-analysis, we also obtained blood from ten women with histologically confirmed endometriosis. We also recruited 19 healthy volunteers at the Royal Women Hospital and Monash University, Australia following informed written consent and ethical approval from the institution’s research board.

The validation phase cohorts were not pre-selected according to inclusion and exclusion criteria that were used in the training phase study. Therefore, 7 out of 25 patients with HGSOC and 5 out 25 women with benign ovarian masses had complex medical co-morbidities such as major cardiovascular diseases, complicated or uncontrolled medical conditions, active autoimmune disease as well immune suppressing medications (Supplementary Table 1). All relevant clinical information, including age, self-reported menopausal status, pre-existing conditions or medications, any prior history of malignancy, comprehensive histological assessment of tumour type, stage and grade by qualified gynaecological oncologists were obtained from de-identified patient medical records. Venous blood samples were obtained from anaesthetized patients prior to any surgical or chemotherapy treatment. Measurement of serum CA125 for all study patients was performed in the diagnostic pathology laboratory, either at the Monash Medical Centre or Royal Women Hospital, Melbourne, Australia.

### Serum and plasma isolation

Serum and plasma were isolated from whole blood collected either in serum separation tubes (primary cohort) or EDTA-coated tubes (validation study) by centrifugation at 3000 rpm for 10 minutes. All blood samples were separated within less than 3 hours of being obtained. After removing cellular and protein debris, serum/plasma was aliquoted and stored at −80 °C until later use. Blood parameters i.e. hemoglobin (Hb), platelet count (PLT), white cell count (WCC), absolute neutrophil count (ANC), total lymphocyte count (TLC) and serum CA125 were routinely determined in all patients suspected of ovarian cancer and prior to surgery.

### Multiplex bead immunoassays

#### Training phase

Multiplex magnetic bead immunoassay kits were used as per manufacturer’s protocol (Invitrogen) to simultaneously measure 28 numerous analytes in a single sample. Human 25-Plex panel was used to determine quantitative measurement of cytokines (GM-CSF, IFN-α, IFN-γ, IL-1β, IL-1RA, IL-2, IL-2R, IL-4, IL-5, IL-6, IL-7, IL-8, IL-10, IL-12 (p40/p70), IL-13, IL-15, IL-17 and TNF-α) and chemokines (RANTES, Eotaxin, CCL2/MCP-1, CCL3/MIP-1alpha, CCL4/ MIP-1beta, CXCL9/MIG, CXCL10/IP-10) in study serum. This 25-Plex panel was combined with singleplex cytokine beads sTNFRII and CCL22/MDC purchased separately. TGF-β was analysed separately using a singleplex bead kit. HE4 was assayed using R&D human pre-mixed magnetic Luminex assay kit.

#### Validation phase

For the validation study, a 6-panel kit consisting of IFN-γ, IL-1RA, IL-6, IL-10, TNF-α, sTNFRII was used and TGF-β and HE4 were assayed as a singleplex kit.

Both the training and validation phases were analysed using the same assay protocol. Prior to analysis, serum samples were completely thawed, clarified by centrifugation (1000 g for 10 mins) and filtered to prevent clogging of the filter plates. Serum samples for TGF-β assay required acid-treatment and neutralization to remove latency associated peptide from TGF-β1 prior to use in assay. The serum samples were randomly assigned to the plates to avoid assay bias and were analysed in duplicates to determine inter-assay differences. A filter-bottom, 96-well microplate was coated with 25 μl antibody-coated beads. A standard curve was made by serially diluting the human cytokine standard cocktail in assay diluent. Standards were pipetted at 100 μl per well in duplicates, while patient sera were pipetted at 50 μl per well in duplicates. 50 μl of assay diluent was added to the standards and patient samples, and 50 μl of incubation buffer was added to all wells. The plates were incubated for 2 hours at room temperature on an orbital shaker at 500–600 rpm in the dark at room temperature. Wells were washed twice using a magnetic separator. A cocktail of biotinylated secondary antibodies was added, and the microplate was incubated for an hour in the dark on a microtiter shaker. Following twice washing with magnetic separator, streptavidin-phytoerythrin was added and incubated under agitation for 30 minutes in the dark at room temperature. The plate was washed for three times and 100 μl of wash solution was added to each well and the samples were analysed using Luminex® 200TM analyser (Luminex Corp) as per standard protocol. For each analyte, 100 beads were analysed and the median fluorescence intensity was determined. Analysis of median fluorescence intensities was performed using five-parameter logistic curve fitting to the standard analyte values. The inter-assay variability of each assay was 2% to 8% and the intra-assay variability was 2% to 7%.

### RMI calculation

The RMI (Risk of Malignancy index) score was calculated based on menopausal status (M), serum CA125 concentration and ultrasound scores (U) for all patients. The equation used:

$$RMI={Ux}\,{Mx}\,{CA125}\,{concentration}\,{in}\,{plasma}\,{or}\,{serum}$$

where: M-menopausal status-(1 = premenopausal, 3 = postmenopausal), U-ultrasound scores- features include bilateral mass, presence of solid areas, multi-loculated cyst, intra-abdominal metastases and ascites^10^. Each feature assigned a score 0, 1 or 3. (0 = imaging score of 0, 1 = imaging score of 1, 3 = imaging score of 2–5). **CA125** – the actual concentration in IU/ml is used.

### ROMA calculation

ROMA (Risk of Malignancy algorithm) was calculated using biomarkers HE4 and CA125 based on logistic regression formula developed by Moore *et al*.^22^:

$$\begin{array}{rcl}{\rm{Premenopausal}}:{\boldsymbol{Predictive}}\,{\boldsymbol{Index}}({\boldsymbol{PI}}) & = & {\bf{12}}.{\bf{0}}+2.38\ast \mathrm{LN}({\rm{HE}}4)\\  &  & +\,{\bf{0}}.{\bf{0626}}\ast \mathrm{LN}({\boldsymbol{CA}}{\bf{125}})\\ {\rm{Postmenopausal}}:{\boldsymbol{Predictive}}\,{\boldsymbol{Index}}({\boldsymbol{PI}}) & = & -{\bf{8}}.{\bf{09}}+1.04\ast \mathrm{LN}\,({\rm{HE}}4)\\  &  & +\,{\bf{0}}.{\bf{732}}\ast \mathrm{LN}({\boldsymbol{CA}}{\bf{125}})\\ ROMA\,score( \% ) & = & [\frac{\exp (PI)}{1+\exp (PI)}\ast 100]\end{array}$$

### Statistical analysis of data

As this was an exploratory, observational study, there was no formal, documented statistical analysis plan. The research team agreed that a variety of parametric and non-parametric approaches (for supervised learning) would be applied to one data set to detect a signal and that a second data set would be used to check, or validate, the learning.

Table 2 The continuous variable of three groups were analysed primarily using non-parametric Kruskal-Wallis analysis followed by secondary analysis using Dunn’s multiple comparison test in GraphPad Prism. The results were reported in mean ± standard deviation (SD). For categorical variables involving three groups, primary analysis was performed using Freeman-Halton extension of Fisher’s exact test to compute the (two-tailed) probability of the distribution of the categorical values in 3 × 2 or 3 × 3 contingency table using Graphpad Prism.

**Figure 1:**
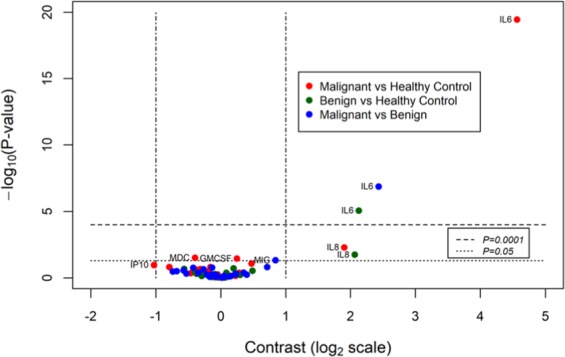
Volcano plot of soluble factors in pre-operative sera. Pre-operative blood was withdrawn from 33 patients with ovarian cancer, 15 benign ovarian mass and 21 with normal ovaries. A total of 28 serum soluble factors were measured using multiplexed bead immunoassay. One-way analyses of variance were conducted on each of the 28 log_2_-transformed immune factors. For each immune factor, the three pairwise contrasts of means, malignant tumour versus healthy control, benign tumour versus healthy control and malignant tumour versus benign tumour, and their standard errors of differences, were calculated and the results of the pairwise t-tests were summarized in a volcano plot of statistical significance, −log_10_(*P*-value), versus fold change, namely the difference in the group means of the log_2_-transformed soluble factors concentrations. Volcano plot of statistical significance (−log_10_(*P*-value) of contrasts versus contrasts expressed as differences on the log2 scale. Soluble factors with contrasts greater than a 2-fold change or with a *P*-value < 0.05 are labelled.

For Fig. 1 One-way analyses of variance were conducted on each of the 30 log_2_-transformed immune factors including CA125 and HE4 (Interleukin-17 was not statistically analysed as all values were below the detection limit and was excluded from analysis). For each immune factor, the three pairwise contrasts of means, malignant tumour versus normal/healthy control, benign tumour versus normal/healthy control and malignant tumour versus benign tumour, and their standard errors of differences, were calculated and the results of the pairwise t-tests were summarized in a volcano plot of statistical significance, −log_10_(*P*-value), versus fold change, namely the difference in the group means of the log_2_-transformed cytokine concentrations. For each analysis of variance, diagnostic residual plots, including normal and half-normal probability plots, were examined to check the assumptions on which the analysis was based (viz homogeneity of the variance, and normality, of the residuals). Figure 1 code attached CodeForFiguresandTables.docx

**Figure 2:**
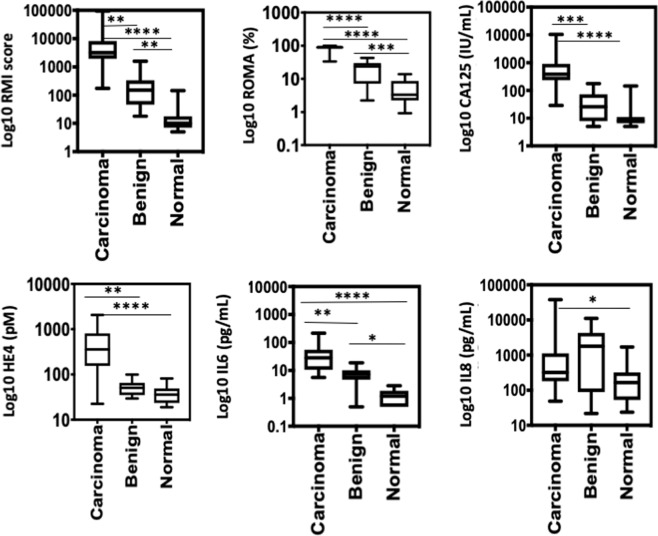
RMI score, ROMA and serum level of pro-inflammatory cytokines in advanced serous EOC patients and those with benign ovarian masses and normal ovaries in training cohort. Box-and-whisker-plots extending down to the minimum value and up to the maximum level of RMI score, ROMA, serum levels of CA125, HE4, IL-6 and IL-8 in patients with high-grade serous EOC, with benign ovarian masses and normal ovaries. The p-value is indicated for each factor. Kruskal-Wallis followed by Dunn’s multiple comparison test: *p < 0.05, **p = 0.001–0.01, ***p = 0.0001–0.001, ****p < 0.0001.

Figure 2 The continuous variable of three groups were analysed primarily using non-parametric Kruskal-Wallis analysis followed by secondary analysis using Dunn’s multiple comparison test using Graphpad Prism. The results were reported in mean ± standard deviation (SD). For categorical variables involving three groups, primary analysis was performed using Freeman-Halton extension of Fisher’s exact test to compute the (two-tailed) probability of the distribution of the categorical values in 3 × 2 or 3 × 3 contingency table using Graphpad Prism.

**Figure 3:**
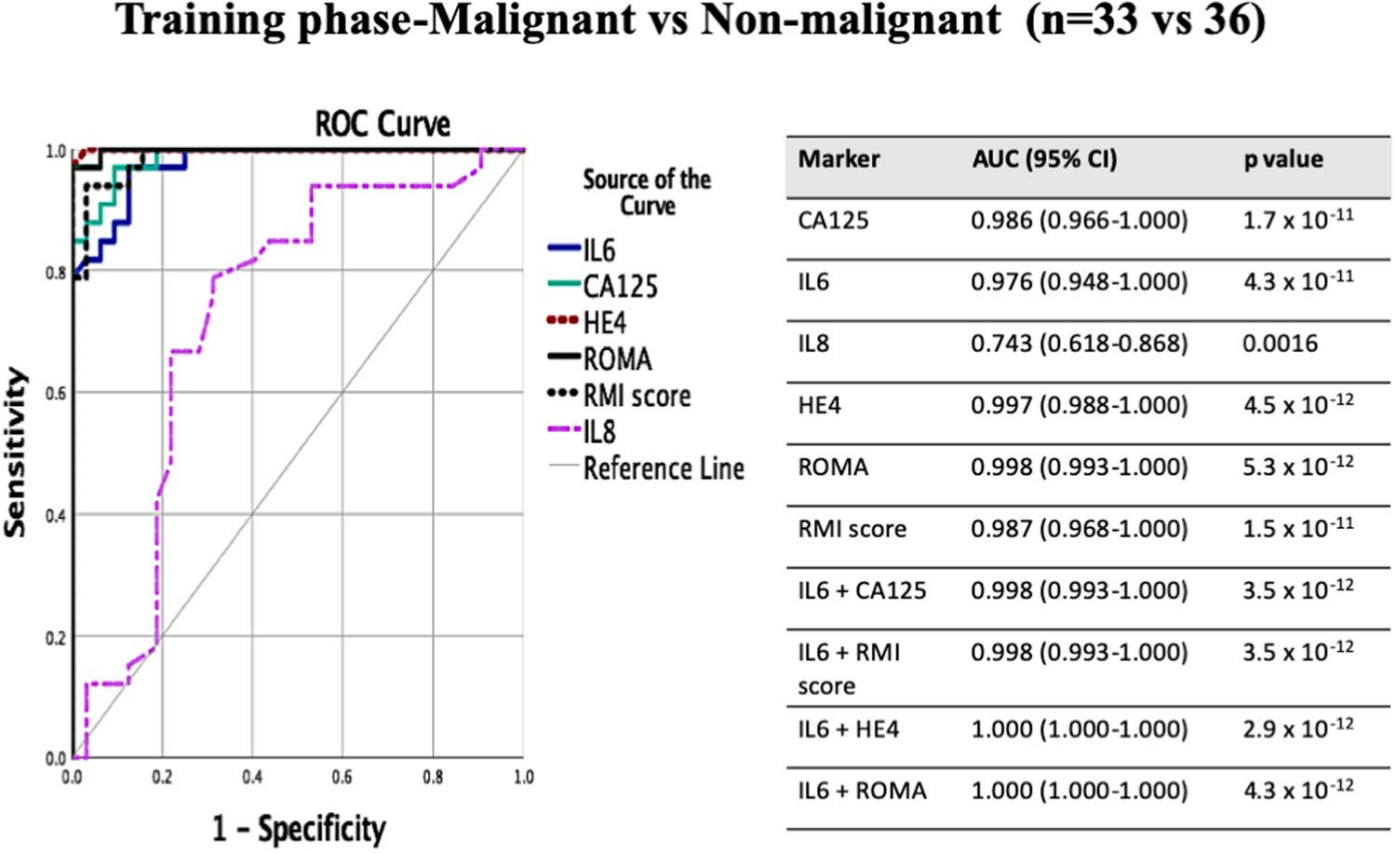
ROC-AUC value using CA125, HE4, IL-6, IL-8, RMI score, ROMA alone of the training phase for patients with malignant and non-malignant ovarian masses. ROC analysis of all markers alone for the training phase and associated tables with predicted probability in combination. ROC-receiver operating characteristic, AUC- area under curve, 95% CI- 95% Confidence Interval. Reference line (grey continuous line) is at AUC 0.5).

**Figure 4:**
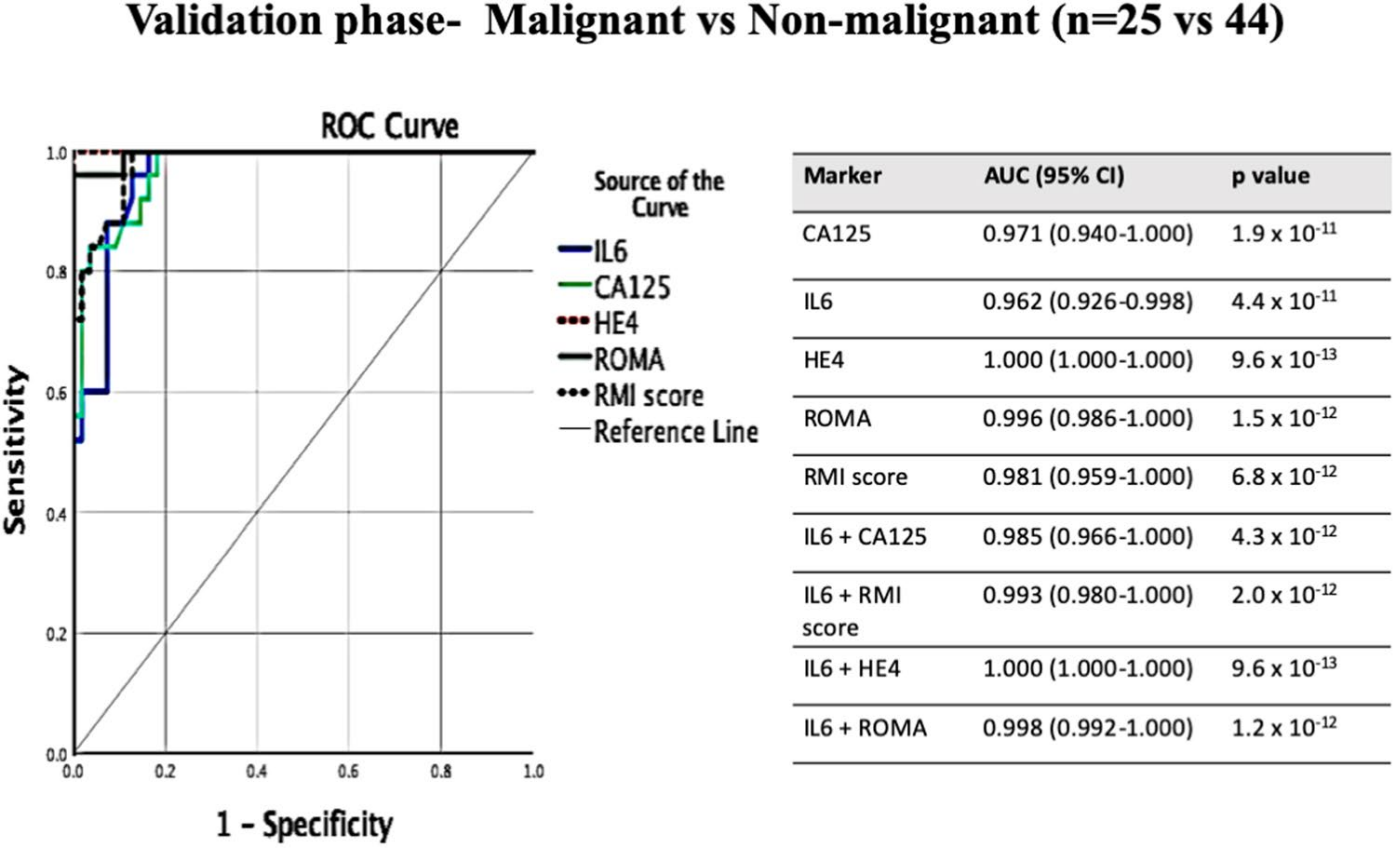
ROC-AUC value using CA125, HE4, IL-6, IL-8, RMI score, ROMA alone of the independent validation phase for patients with malignant and non-malignant ovarian masses ROC analysis for all markers alone for the validation phase and associated tables with predicted probability in combination. ROC-receiver operating characteristic, AUC- area under curve, 95% CI- 95% Confidence Interval. Reference line (grey continuous line) is at AUC 0.5).

Figures 3 and 4 and Table 3 ROC (Receiver operator characteristic) analyses were performed to determine the predictive value of each marker individually or in combination. Area under curve (AUC) was obtained for each analytes and predictive probability for analytes in combination were also determined using binomial logistic regression. Good risk prediction models will have an AUC greater than 0.7^26^, and the most informative biomarker will increase AUC by 0.005 or more^27^. Statistical significance was defined as p < 0.05 where appropriate and 95% confidence interval (CI) was also reported. Figures 3 and 4 codes attached CodeForFiguresandTables.docx

**Table 3:** ROC analysis using CA125, HE4, IL-6, IL-8, RMI score, ROMA alone and predicted probability in combination for distinguishing patients with malignant HGSOC from those with benign ovarian mass, malignant HGSOC from patients with normal ovaries, and benign ovarian mass from patients with normal ovaries in the training phase.

Markers	Malignant vs Benign			Malignant vs Normal			Benign vs Normal		
	AUC	95% CI	p value	AUC	95% CI	p value	AUC	95% CI	p value
CA125	0.96	(0.91–1.00)	3.0 × 10^–6^	0.99	(0.98–1.00)	1.2 × 10^−9^	0.73	(0.53–0.95)	0.027
RMI score	0.97	(0.92–1.00)	2.0 × 10^−6^	1.00	(1.00–1.00)	7.8 × 10^−10^	0.95	(0.88–1.00)	2.3 × 10^−6^
HE4	0.99	(0.99–1.00)	4.0 × 10^−7^	1.00	(1.00–1.00)	7.8 × 10^−10^	0.84	(0.69–0.99)	0.0013
ROMA	0.99	(0.98–1.00)	4.9 × 10^−7^	1.00	(1.00–1.00)	7.8 × 10^−10^	0.84	(0.69–0.99)	0.0013
IL6	0.92	(0.85–1.00)	1.4 × 10^−6^	1.00	(1.00–1.00)	7.8 × 10^−10^	0.91	(0.76–1.00)	1.4 × 10^−6^
IL8	0.64	(0.43–0.84)	0.17	0.72	(0.59–0.86)	0.006	0.72	(0.51–0.93)	0.036
IL6 + CA125	0.99	(0.98–1.00)	4.3 × 10^−7^	1.00	(1.00–1.00)	7.8 × 10^−10^	0.99	(0.96–1.00)	4.0 × 10^−6^
IL6 + RMI score	0.99	(0.99–1.00)	6.4 × 10^−7^	1.00	(1.00–1.00)	7.8 × 10^−10^	0.97	(0.91–1.00)	1.0 × 10^−5^
IL6 + HE4	1.00	(1.00–1.00)	3.7 × 10^−7^	1.00	(1.00–1.00)	7.8 × 10^−10^	0.97	(0.91–1.00)	1.0 × 10^−5^
IL6 + ROMA	1.00	(1.00–1.00)	3.7 × 10^−7^	1.00	(1.00–1.00)	7.8 × 10^−10^	1.00	(1.00–1.00)	8.5 × 10^−6^

ROC-receiver operating characteristic, AUC- area under curve, 95% CI- 95% Confidence Interval.

PPV- positive predictive value, NPV-negative predictive value.

ROC-receiver operating characteristic, AUC- area under curve, 95% CI- 95% Confidence Interval.

Table 4 The diagnostic sensitivity, specificity, positive predictive value (PPV) and negative predictive value (NPV) were assessed by receiver operating characteristic (ROC) curves in SPSS. The accuracy can be defined as the percentage of correctly classified instances (TP + TN)/(TP + TN + FP + FN), where TP, FN, FP and TN represent the number of true positives, false negatives, false positives and true negatives, respectively. Misclassification is calculated as the percentage of falsely classified instances (FP + FN)/(TP + TN + FP + FN).

**Table 4:** Evaluation of IL-6 < 3.75 pg/ml and in combination with conventional biomarkers and tests in the training phase to discriminate between malignant and non-malignant ovarian masses.

Marker	Sensitivity (%)	Specificity (%)	PPV (%)	NPV (%)	Accuracy (%)	Misclassification (%)
CA125	96.3	84.3	81.8	97.0	89.3	10.60
RMI score	96.5	86.4	84.8	97.0	90.9	9.09
HE4	97.1	100	100	97.0	98.5	1.51
ROMA	97.0	91.4	90.9	96.7	93.9	6.06
IL6 + CA125	100	91.7	90.9	100	95.4	4.54
IL6 + RMI score	100	94.2	93.9	100	97.0	3.03
IL6 + HE4	100	100	100	100	100	0.00
IL6 + ROMA	100	94.2	93.9	100	97.0	3.03
IL6 + CA125 + HE4	100	100	100	100	100	0.00

PPV- positive predictive value, NPV-negative predictive value.

**Figure 5:**
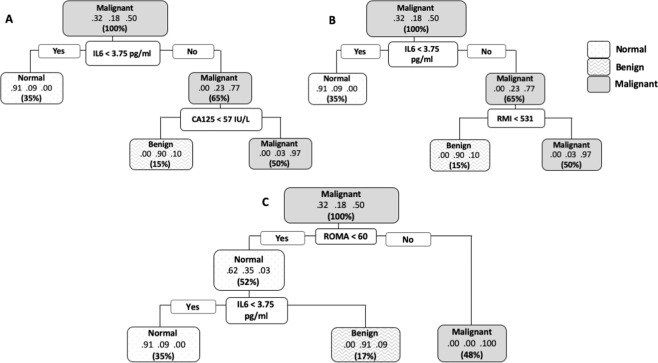
Classification and regression trees (CART) analysis using the recursive partitioning method to evaluate usefulness of IL-6 and components of the conventional risk biomarker CA125 and indices such as RMI score (menopause status, ultrasound score CA125), and ROMA (CA125 and HE4) in discriminating between patients with malignant (n = 33), benign (n = 12) and normal ovarian masses (n = 21) in the training phase. IL-6 levels were evaluated in trees in combination with either CA125 (**A**), RMI (**B**) or the ROMA index. (**C**) The values within each node or subnodes from left to right represent the proportion of each group (normal, benign and malignant) from the total number of patients (N = 66).

For Fig. 5A–C The method of recursive partitioning was used to identify cytokines and components of the RMI and ROMA indices that were associated with the disease status (normal, benign or malignant) of the patients in the training phase. In a second stage of investigations, the most promising cytokines were evaluated in trees that included either the CA125 alone, RMI index or the ROMA algorithm. Three types of tree were evaluated (each type consisting of the combination of relevant cytokines with one of the three conventional indices). For each type, the tree with the minimum value of the Gini index (this is the default in rpart) was selected subject to the constraint of a minimum node size (bucket size) of 10. Sensitivity, specificity, positive predictive value (PPV), negative predictive value (NPV), accuracy and misclassification rate were then calculated for components of conventional tests (RMI and ROMA) and biomarkers (CA125 and HE4) alone or in combination at their cut-off levels used in the clinics with IL-6 level derived from the trees. Figure 5A–C codes attached CodeForFiguresandTables.docx

Supplementary Table 1 The continuous variable of three groups were analysed primarily using non-parametric Kruskal-Wallis analysis followed by secondary analysis using Dunn’s multiple comparison test in GraphPad Prism. The results were reported in mean ± standard deviation (SD). For categorical variables involving three groups, primary analysis was performed using Freeman-Halton extension of Fisher’s exact test to compute the (two-tailed) probability of the distribution of the categorical values in 3 × 2 or 3 × 3 contingency table using Graphpad Prism.

For Supplementary Fig. 1 Principal-component analysis (PCA) was performed to describe the variance of cytokine concentrations using SPSS. All the variables were transformed using log2 to reduce skewness prior to PCA. Resulting PCA component scores were extracted for analysis from overall data. Scree-test and eigenvalues > 1.0 were used to determine the number of factors. The data was examined for intrinsic variation and whether any clustering was presented at this stage. Resulting PCA component scores were extracted for analysis. Secondary analysis was performed to check individual factor score values. All individual scores were plotted on a simple scatter plot, and the individuals were then categorized according to tumour status: malignant, benign and normal/healthy controls. Supplementary Fig. 1 code attached CodeForFiguresandTables.docx

For Supplementary Figs. 2 and 3 ROC (Receiver operator characteristic) analyses were performed to determine the predictive value of each marker individually or in combination. Area under curve (AUC) was obtained for each analytes and predictive probability for analytes in combination were also determined using binomial logistic regression. Good risk prediction models will have an AUC greater than 0.7^26^, and the most informative biomarker will increase AUC by 0.005 or more^27^. Statistical significance was defined as p < 0.05 where appropriate and 95% confidence interval (CI) was also reported. Figures 2 and 3 codes attached CodeForFiguresandTables.docx

Analyses of variance and ROC curve analyses were also conducted on the validation data set. The validation exercise was conducted by using the splitting rules associated with the classification trees that were derived from the training phase, to classify patients in the validation data set and to thereby form predictions of their status. These predictions of the status of the patients were then compared to the actual status of the patients in 3 × 3 contingency tables and Somers’ D statistic was used to assess the agreement between predicted and actual status.

Graphpad Prism version 8.3.0 and SPSS IBM 23.0 were used for analyses of PCA, ROC curve and logistic regressions. GenStat (Version 17.1) was used for analyses of variance and estimation of contrasts of means. The R programming language (Version 3.3.2) was used to create the volcano plot, the rpart library was used to construct classification trees with minimum node size set to 10. The rattle library was used to draw the resulting trees. The SAS package (Version 9.4) was used to calculate Somers’ D.

## Results

### Training phase

All patients with ovarian cancer had Stage III-IV high-grade serous adenocarcinoma (n = 33) and the majority of patients with a benign ovarian mass (n = 12) had a serous cystadenoma (75%). No ovarian pathology was found in the patients undergoing risk reduction surgery for a genetic mutation or family history of hereditary breast or ovarian cancer (n = 21). The median age of patients with ovarian cancer and those with benign ovaries were 60 and 55 years respectively, while the median age for patients with normal ovaries was younger at 48 (Table 2). There was no significant difference in the haemoglobin, platelet and white cell count and absolute neutrophil count level between the three groups of patients. The mean of total lymphocytes was significantly lower in ovarian cancer patients compared with those with benign ovarian mass (Table 2) and normal ovaries.

Figure 1 summarizes all 28 immune soluble factor concentrations measured using multiplex bead immunoassay analysed using one-way analyses of variances. The pair-wise t-tests and contrast of means between the three groups (malignant, benign and normal ovaries) were calculated for each immune soluble factor and summarized in the volcano plot analysis (Fig. 1). Immune factors IL-6 and IL8 in the pre-operative sera emerged as the only two cytokines with greater than a 2-fold change. Serum IL8 concentrations were more than 2-fold different between patients with malignant and normal ovaries as well as between benign and normal ovaries (p < 0.05). Serum IL8 concentrations were not statistically different between patients with malignant and benign ovarian masses. Pre-operative IL-6 concentrations differences were greater than 2-fold in all three groups: malignant versus benign, benign vs normal and malignant versus normal ovaries (p < 0.05) (Fig. 1).

All collected blood factors and clinical parameters were further included in a PCA analysis. Given the age difference between patients with malignancy and normal ovaries, age as well as menopausal status were further incorporated as factors in the PCA. Four components with eigenvalues above one were extracted, indicating that they were the main contributors to the variability of the samples. Component 1 represented 15.8% of total variances of the study groups. Component 1 included the conventional diagnostic tools and biomarkers, RMI score, ROMA, HE4 and CA125, as well as the immune factors IL-6 and IL8 (Supplementary Fig. 1).

These six factors were individually assessed for their ability to distinguish between the three groups of patients using Kruskal-Wallis analysis, followed by Dunn’s multiple comparison test (Fig. 2). The median of RMI score in patients with HGSOC was significantly higher compared to those with benign ovarian masses and normal ovaries (3204 vs 151 vs 10 IU/mL, p = 0.001). Similarly, the mean of ROMA in patients with HGSOC was also significantly higher percentage at 88.4 ± 12.4% median 89.3, range: 33.6–99.7%) compared to those with benign ovaries (19.9 ± 13.4, median 23.78%) and normal ovaries (3.33%).

The patients with ovarian cancer had a higher mean of serum CA125 level (1125 ± 2270, median 372, range: 29–10430 IU/mL) compared to those with benign masses (47.8 ± 55.1, median:26, range: 5–177 IU/mL) and normal ovaries (15.1 ± 29.9, median 8.0, range 5–145), adjusted p = 0.0002 and 0.0001 respectively. The serum levels of CA125 were not able to discriminate between those with benign and normal ovaries, (adjusted p value following Dunn’s post-hoc test was 0.60). Although the median of CA125 levels among those in benign group was observed to be twice as high as those with normal ovaries, the adjusted p value was not significant at 0.60. This may be largely attributed to the small sample size of the benign group.

The mean HE4 level in patients with HGSOC was significantly higher (583 ± 589, median 357.4, range: 22.4–2060 pM) compared to those with benign ovaries (54.3 ± 23.5, median 50.6, range: 29.3–99.3 pM) and normal ovarian mass (38.4 ± 16.3, median 35.7, range: 18.9–81.3 pM). Following Kruskal-Wallis analysis and multiple comparisons with Dunn’s test, serum HE4 was able to discriminate ovarian cancer from those with benign masses, as well with normal ovaries. Similar to CA125, the level of HE4 was not able to discriminate between benign and normal ovaries with adjusted p value of 0.84.

The median level of IL-6 was higher in patients with HGSOC compared to benign ovarian masses or normal ovaries (28.3 vs 7.4 vs 1.2 pg/ml, p = 0.0001) (Fig. 2). Using multiplex magnetic bead immunoassay kits, the level of 28 analytes, including IL6 was measured. We found that the level of IL-6 was below the level of detection in the serum of 28.5% (6/21) patients with normal ovaries in this study, while 6.7% (1/15) of patients with a benign ovarian mass and no patients with Stage III-IV HGSOC had an undetectable level. The majority of patients (97%) with Stage III-IV HGSOC had elevated IL-6 with a mean concentration of 40.4 ± 40.6, range: 5.6–215.88 pg/ml compared to those with benign masses (6.8 ± 3.0, range: 0.5–10.73 pg/ml and normal ovaries (1.3 ± 0.7, range 0.5–2.8 pg/ml), which was statistically significant following Kruskal-Wallis and Dunn’s post-hoc analysis (Fig. 2). Therefore, IL-6 by itself could distinguish between the three study patient groups. A total of eighteen out of 33 (54.5%) patients had ascites in the training cohort. Significantly higher IL-6 levels were detected in patients’ ascites (median, 18,050 pg/ml [range: 5162 to 122,883 pg/ml]) compared with serum (median, 53 pg/ml [range: 11.2 to 216 pg/ml]) (p = 0.0001). The presence of ascites did not correlate with higher levels of serum IL6 (p = 0.09).

The mean serum IL8 concentration in patients with ovarian cancer (3124 ± 7336, median 320.5, range: 48.6–37574 pg/ml) were significantly higher when compared to healthy controls (262.4 ± 368.6, median 165.7, range: 23.7–1694 pg/ml, p = 0.02). Serum IL8 was not able to distinguish between benign and malignant ovarian masses as elevated concentrations of IL8 were also observed in benign masses (1961 ± 2400, median 1363, range: 21.8–7965 pg/ml), p = 0.07. Although the median of IL8 levels among those in benign group is observed to be 1.5 times higher than those with normal ovaries, the adjusted p value following Dunn’s post-hoc test was not significant. This may be largely attributed by the small sample size of the benign group. None of the other factors analysed could distinguish between the three study groups.

As RMI score and ROMA are the current diagnostic tests used in the prediction of malignancy to facilitate referral to specialized center, ROC (Receiver operator characteristic) analysis which combines the strengths of sensitivity and specificity was performed to calculate the area under curve (AUC), which summarizes overall test performance^28^ (Fig. 3). The value of AUC was used to evaluate the performance of selected factors for the ability to discriminate between the groups (malignant vs benign, malignant vs normal and benign vs normal). The value closer to one indicates better discriminatory test performance, while AUC = 0.50 would suggest predictive accuracy equivalent to that of chance alone^29^. IL8 had an AUC value of 0.743 (95% CI 0.618–0.868), which show acceptable predictive ability to discriminate malignant and non-malignant patients but was not superior or comparable to AUC value of CA125 (0.986), HE4 (0.997), RMI score (0.987) or ROMA (0.998) (Fig. 3). By contrast IL-6 showed excellent predictive value in distinguishing between malignant and non-malignant patients (AUC 0.976, 95% CI 0.948–1.000), comparable to the AUC value for conventional tests (RMI and ROMA) and biomarkers (CA125 and HE4) (Fig. 3A). The AUC value was also calculated for all immune factors and conventional tests between the three study groups: HGSOC vs benign ovarian mass, malignant vs normal ovaries and those with benign ovarian mass vs normal ovaries (Table 4). IL-6 alone (AUC 0.927) had a good predictive AUC value, similar but not superior to conventional tests and biomarkers to distinguish between malignant and benign ovarian patients (Table 3). The predictive ability of IL-6 was excellent (AUC 1.000) when it came to be discriminating between those with malignant and normal ovaries (Table 3). Furthermore, IL-6 (AUC 0.905) was superior to CA125 (AUC 0.73), HE4 (AUC 0.84) or ROMA (AUC 0.84) in distinguishing between patients with a benign ovarian mass and normal ovaries (Table 4). Moreover, the combination of IL-6 with existing conventional diagnostic tools such as RMI score and ROMA or conventional biomarkers CA125 and HE4 improved the ability of the tests to identify patients with malignant ovaries (Table 3, Figs. 3,4).

To evaluate the useful thresholds for combining IL-6 and conventional tests to discriminate disease status (normal, benign or malignant), classification and regression trees (CART) were fitted to the data in the training phase using recursive partitioning method. The analyses identified a serum concentration of IL-6 of less than 3.75 pg/ml as informative for use as initial triage to identify patients with normal ovaries. In the three trees (Fig. 5A–C) selected for further investigation, 21/21 normal patients were correctly assigned to the ‘normal’ group at this threshold. In a second stage of the CART analyses, IL-6 was evaluated in trees in combination with either CA125, RMI index or ROMA index. IL-6 at less than 3.75 pg/ml correctly identified all patients with normal ovaries as normal in all trees (Fig. 5A–C). Inclusion of CA125 or RMI (at the thresholds resulting from CART analysis of the same dataset), into the IL-6-tree resulted in a decrease in the misclassification rate of CA125 or RMI alone (Table 4). Incorporating HE4 and ROMA into the IL-6-tree also improved the accuracy of discrimination of malignant disease, with the highest accuracy and zero misclassification rate seen in the combination of HE4 and IL-6, and/or CA125 (Table 4). This system may offer therefore a more robust classification, with improved accuracy and lowered misclassification rates than observed for current methods as a result of the inclusion of IL-6 as demonstrated in this study (Table 4).

### Validation phase

To evaluate the usefulness of the three IL-6-based classification, plasma samples from an independent cohort, including 25 patients with advanced stage high grade serous ovarian cancer, 25 patients with a benign ovarian mass and 19 healthy volunteers, were tested. The pre-treatment samples from this cohort were collected prospectively from 2014 and stored in a biobank till date of assay. The demographic characteristics of the second cohort are summarized in Supplementary Table 1. In the cancer group, all patients with ovarian cancer had high-grade serous adenocarcinoma (HGSOC) (n = 25) and majority of patients with a benign ovarian mass (n = 25) had serous cystadenoma (60%). Healthy volunteers were recruited as the study control group (n = 19). The mean age of patients with ovarian cancer and those with a benign ovarian mass were similar, while healthy controls were relatively younger (Supplementary Table 1). In the validation cohort, a total of thirteen out of 25 (52.0%) patients had ascites. Similar to the training cohort, the presence of ascites did not correlate to higher levels of serum IL6 among the patients with HGSOC (p = 0.19).

Pre-operative plasma in the validation phase cohort was analysed selectively for relevant cytokines including CA125 and HE4. PCA analysis of all demographic, blood components and immune factors, similar to the training cohort, revealed IL-6 as the one factor that clustered with RMI score, ROMA, CA125 and HE4 in component 1 which represented 32.4% of total variances in this study group (data not shown). These factors were then assessed for their discriminative ability between the three study groups using one-way ANOVA, followed by Dunn’s multiple comparison test as post hoc (Supplementary Fig. 2). As observed in the training study, in this validation cohort, IL-6 and RMI, but not ROMA, remained discriminative markers for all three study groups. Plasma CA125 and HE4, similar to the training phase, displayed similar discriminative ability and were not able to distinguish between patients with a benign ovarian mass and healthy controls (Supplementary Fig. 2).

IL-6 retained good predictive value in the validation phase by ROC analysis and was clearly able to distinguish malignant ovarian patients from their non-malignant counterparts (AUC 0.962, 95% CI 0.926–0.998, p = 4.4 × 10^−11^). The combination of IL-6 with conventional tests such as RMI score and ROMA or conventional biomarkers such as CA125 or HE4 similarly improved the predictive probability of malignant ovarian masses in the validation phase (Fig. 3). Comparison of the ROC curves of IL-6 alone or in combination with conventional tests and biomarkers between the three study groups in the validation phase revealed a similar pattern to training phase (Supplementary Table 2).

The patients in the validation phase were then classified by applying the splitting rules derived from the classification trees of the training phase dataset to enable predictions of their status. The predicted status of the patients was then compared to the actual status of the patients. Inclusion of IL-6 to the trees correctly classified all 19 healthy control patients as normal, essentially eliminating false positives prior to incorporation of conventional tests and biomarkers. Of the three trees, the classification tree based on IL-6 and CA125 was the best (Somers’ D = 0.943 ± 0.028) with 3 out of 25 malignant patients classified as benign and there were no false positives. In comparison, when CA125 is used alone, there were 2 cases of false positives. Similarly, but only marginally better, was the tree based on IL-6 and RMI (Somers’ D = 0.929 ± 0.030) while the classification tree based on IL-6 and ROMA performed poorly (Somers’ D = 0.607 ± 0.071) when it was tested on the validation data set. This reduction in goodness of fit, which was most evident in the tree that was based on IL-6 and ROMA, is more likely an example of the general phenomenon of “over fitting” rather than multiplicity. Invariably a statistical model constructed from one set of data does not perform as well on a new set of data. However, we believe we have demonstrated that the classification tree based on IL-6 and CA125 performs very well.

We also assessed the usefulness of IL-6 in helping to discriminate ovarian cancer patients. One of the features of endometrioma is the elevation of CA125 concentration, which may be misleading or reduce the capacity to discriminate endometrioma from ovarian cancer. We obtained sera from ten patients with benign endometrioma and measured CA125 concentration and relevant cytokines including IL-6. The mean CA125 concentration was 109.3 ± 128.8 IU/L (median 81, range: 7.5–436 IU/L) which was 7-fold higher than the mean concentration of a serous cystadenoma 20.8 ± 24.0 IU/L (median 17, range: 7.0–129 IU/L), p = 0.009. However, the mean IL-6 concentration (1.33 ± 0.90, median 1.2, range: 0.5 to 3.71 pg/ml) in the plasma for these ten patients with endometrioma remained comparable to the mean concentration of IL-6 of patients with benign serous cystadenomas (1.15 ± 0.52, median 1.4, range 0.5 to 2.79 pg/ml), p = 0.16. The concentration of IL-6 in the endometrioma group was still significantly lower by 6-fold compared to IL-6 concentration in the malignant diseases (p = 1.0 × 10^−6^). All concentration levels among women with endometrioma were also lower than the threshold point of 3.75 pg/ml (Supplementary Fig. 3).

## Discussion

The present study shows for the first time that IL-6, a pro-inflammatory cytokine, overexpressed in sera or plasma of advanced high-grade serous ovarian cancer patients, is the single most informative cytokine out of 28 soluble factors able to enhance the diagnostic efficiency of conventional tools (RMI and ROMA) and biomarkers (CA125 and HE4) to discriminate between women with a malignant ovarian mass, benign ovarian pathology and healthy ovaries.

IL-6 had overall good predictive value to discriminate malignant ovarian masses in both training and validation cohorts, which was comparable to pre-existing current diagnostic tools- CA125, HE4, RMI score and ROMA. Specifically, in this study, IL-6 was an excellent predictive marker for distinguishing between advanced malignant and normal ovaries (AUC 1.000, p = 7.8 × 10^−10^).

In the present study, the median IL-6 level was 24-fold higher in advanced ovarian cancer patients than those with normal ovaries. The IL-6 level remained at a highly detectable rate in all patients with advanced ovarian cancer, while the level was undetectable in 23.8% of patients with normal ovaries in the training phase. While IL-6 can be produced by human ovarian epithelial cells during follicle development, the levels may not reach the detection threshold^30^. In contrast, excessive levels of circulating IL-6 are observed in advanced ovarian cancers and this may be contributed by either tumour cells or peritoneal mesothelial cells, both of which are major contributors to IL-6 production within the tumour microenvironment^31,32^. IL-6 in the malignant ascites, serum and plasma of patients with advanced ovarian cancer have been shown to correlate positively with advanced disease and poor survival^33–36^. The current study has demonstrated the diagnostic and predictive value of serum IL-6 alone and in combination with other markers in discriminating between malignant and non-malignant ovarian masses.

Past studies have reported higher levels of IL-6 in the serum of patients with ovarian cancer^37,38^ and have incorporated it in complex multi-marker panels for the detection of ovarian cancer^27,39^. To the best of our knowledge, however, this is the only study analyzing the diagnostic value of blood IL-6 singly and in combination with the conventional tests (RMI and ROMA) and biomarkers (CA125 and HE4) in advanced HGSOC patients.

IL-6 also had superior predictive value than CA125, HE4, and ROMA in distinguishing between the benign and normal ovaries group. Although CA125 (at a threshold of >35 U/ml) has not achieved satisfactory sensitivity (81%) and specificity (75%) for ovarian cancer detection^6,40^, CA125 had been reported to have the highest sensitivity for serous subtypes and advanced stages^41^, which was also evident in this study. In this study population, the sensitivity and specificity of serum CA125 level to discriminate between malignant and non-malignant masses were 96.0% and 79.5% respectively for the training cohort, while the validation group had a sensitivity of 97.0 and specificity of 81.8%. The higher sensitivity and specificity of serum CA125 level observed maybe attributed to advanced stages of ovarian cancer and a higher percentage of post-menopausal women compared to those pre-menopausal. The training cohort consisted of twenty-four (36.4%) pre-menopausal and 42 (63.6%) post-menopausal women, while the validation group had 24 (34.8%) premenopausal and 45 (65.2%) post-menopausal women.

In the present study, IL-6 but not CA125, could distinguish between all three study groups in both the training and validation phase cohort. The median IL-6 levels in the training phase were observed to be 4.5-fold higher in benign ovaries compared to normal ovaries respectively. The use of CA125 in combination with IL-6, lowered false positive rates and achieved higher predictive values compared to CA125 alone. A similar pattern of improvement was also observed for RMI, HE4 or ROMA in combination with IL-6.

However, IL-6 alone was not superior to the predictive value of other conventional biomarkers and tests in distinguishing benign and malignant ovarian masses. Serum CA125 or RMI when used alone had higher false positive rates^41^. The combination of IL-6 and CA125 may be particularly useful in distinguishing ovarian endometrioma with falsely elevated CA125 from malignant disease. Comparable serum IL-6 levels were previously reported in women with and without endometriosis^42,43^, while other investigators have reported elevated levels of IL-6 in endometriosis patients compared with normal ovaries^44,45^. Although endometrioma may have elevated levels of IL-6, as observed in our pilot data, the serum IL-6 levels in women with endometrioma were lower than in women with ovarian cancer, in keeping with Darai *et al*.^37^. Therefore, the addition of IL-6 to CA125 may be clinically helpful to distinguish endometrioma compared to use of CA125 alone; this however warrants evaluation in a larger cohort.

In our study, HE4 alone achieved good overall predictive value for patients with ovarian cancer in both the primary (AUC 0.997) and validation (AUC 0.889) cohorts. However, we found that HE4 had lower predictive value in comparison to CA125, when used as a discriminator between benign ovarian mass and normal ovaries, with AUC of 0.706 for the primary cohort and 0.497 for the validation cohort. The use of HE4 alone as a biomarker to distinguish between benign and normal ovaries therefore may not be reliable as shown in previous studies^15,46^. The introduction of ROMA which incorporates CA125 and HE4 has been promising as a more specific discriminator of the malignant ovarian mass^22,47^. In a prospective evaluation of pre-operative ovarian masses in an Australian cohort, ROMA was found not to be inferior to RMI in the detection of ovarian malignancy^47^. Similarly, in our study, ROMA had a good overall predictive value in both the primary (AUC 0.998) and the validation (AUC 0.996) study groups and was therefore a better predictive tool than HE4 alone or the RMI score in our study. In addition, the addition of IL-6 to ROMA enhanced the predictive probability of ROMA to achieve an AUC of 1.000 between all three study populations in this study. The addition of IL-6 to CA125 and HE4 achieved an excellent sensitivity and specificity with the highest accuracy, and therefore may have a potential use in the pre-operative assessment for suspicious ovarian masses in less developed countries with poor access to imaging modalities.

There are limited studies available that might be used to define cut-off values for IL-6 as a diagnostic tool^48^. We found a good discriminatory value for IL-6 at a threshold of >3.75 pg/ml with a sensitivity of 100%, specificity of 76.8%, positive predictive value of 69.7%, and negative predictive value of 100% in this study. This is an improvement to moderate sensitivity/ specificity characteristics reported for IL-6 (sensitivity of 61%, specificity of 79%, positive predictive value of 70%, and negative predictive value of 61%) by Tempfer *et al*.^49^ The difference may be explained by the selection of the study cohort used and the different threshold level for IL-6 being used compared to the current study. While we recruited a homogenous group of advanced FIGO stages (III-IV) HGSOC and compared this with benign cystadenoma and patients with normal ovaries, the previous study included all FIGO stages and various subtypes of ovarian cancer and the control group was healthy blood donors. The threshold level for IL-6 was also lower (0.78 pg/ml) when compared to our study (3.75 pg/ml). In both the training and validation cohorts, we have tried to include a similar age cohort for the control group with age ranging between 40 to 84 years old, however the control group were significantly younger compared to the cancer patients. As the levels of IL6 have been reported to be increased in older women, this data may have potential bias in regard to the threshold level of IL6. Future studies with age-matched control may need to be undertaken to validate the threshold level of IL6.

As cytokines are part of the immune system, which respond rapidly to both external and internal stimuli, cytokine measurements are highly sensitive and are subject to clinical applicability, reproducibility and quality assurance criteria to minimize result variation^24^. The strength of this study is that we recruited a homogenous ovarian cancer population where all of the women had advanced (Stage III-IV) high-grade serous ovarian / fallopian tube cancer and the benign group were mainly patients with a serous cystadenoma, with patients in the control group having their ovaries removed as part of risk reduction surgery. The training phase had a pre-selected population with exclusion of clinical conditions that might have interfered with levels of circulating cytokines. The concentration of IL-6 is largely affected by exercise, weight, stress, significant medical conditions and immunosuppressive drugs^24^. Sample collection and storage may also influence cytokine measurement, and this can affect the validity of the test. Sera samples were collected prior to surgical intervention. The use of lithium heparin and sodium citrate were avoided as it can decrease the measured levels for pro-inflammatory cytokines including IL-6 and TNF^50^. In contrast, the patients recruited to the validation phase were not screened according to the inclusion and exclusion criteria in the training phase study. Therefore, these patients had complex medical co-morbidities such as major cardiovascular diseases, complicated or uncontrolled medical conditions, active autoimmune disease as well as taking immune suppressing medications. This cohort which may more closely reflect the true population encountered during clinical screening, not only allowed validation of IL-6 as a potential biomarker, but also likely demonstrated the true potential of IL-6 as a diagnostic biomarker.

The study is consistent with findings from Nowak *et al*., that patients with advanced ovarian cancer had higher level of pro-inflammatory cytokine IL6 in the serum. Thus, the result of our study supports the critical role of inflammation in ovarian cancer development and progression. As IL-6 plays a substantial role in promoting cancer cell proliferation^24^, elevated concentration of IL-6 are observed not only in association with primary epithelial ovarian cancers, but also in a variety of other solid tumors including breast, lung and colorectal cancers, which can make diagnosis particularly challenging^24,51,52^. The possibility of metastatic breast, lung or bowel cancers merits consideration in the differential diagnosis for a woman with a suspicious ovarian mass and increased IL-6 concentration.

The potential use of peripheral blood IL-6 concentration as a screening tool is promising with IL-6 levels found significantly elevated in early stages (FIGO Stage I and II) of ovarian cancers compared to benign ovarian masses or healthy controls^39,53^. The use of IL6 may improve preoperative discrimination of suspected ovarian tumours, however the findings in this study alone are insufficient to support it for generalized ovarian screening. Given the small number of true early-stage, high-grade serous ovarian cancers available, it was necessary to make inferences using advanced disease cases. Inclusion of early stages of epithelial ovarian cancer and comparison with benign masses and normal ovaries in a larger stage-stratified cohort will be required to ascertain the role of IL6 in generalized ovarian cancer screening including multiple and earlier disease stages.

As with any exploratory, observational study, there is a risk of “over-fitting” a predictive model to the observed data, however inclusion in the study of a second data set to check the usefulness of the model goes some way to alleviating this risk, and we found that IL-6 was still able to accurately identify and triage all 19 normal patients as normal in the validation study. The addition of IL-6 to CA125 or to RMI was further validated as improving their diagnostic utility. Albeit, the analyses generated in the training cohort can be further optimized to specifically account for co-morbidities, given exclusion of these confounders resulted in an increased of ROC-AUC value from 0.962 to 0.985 and 0.993 respectively (Figs. 3 and 4) for the capacity of IL-6 in the validation phase to distinguish between malignant and non-malignant ovarian mass. These findings therefore should be considered when using IL-6 in clinical screening as these will be potential confounders that may influence the levels of IL-6.

## Conclusion

IL-6 alone may be a clinically reliable biomarker to distinguish between patients with advanced high-grade serous ovarian carcinoma and those with normal ovaries. In combination with RMI score, ROMA, HE4 or CA125, IL-6 may enhance the predictive power of existing conventional tools or biomarkers in distinguishing between patients with malignant and benign ovarian masses as well as those with a benign ovarian mass and normal ovaries. Patients with cysts incidentally identified on imaging could potentially be spared surgical intervention. These results indicate that IL-6 may be a useful diagnostic tool for the pre-operative assessment of suspicious ovarian masses. These results support further studies, including those including earlier stages of the disease, to explore and expand on, the potential utility of adding IL-6 to routine diagnostic tests.

## Supplementary information

Supplementary Figures.
