# Supplementary material for: Pre-operative sera interleukin-6 in the diagnosis of high-grade serous ovarian cancer
Source: Sci Rep. 2020 Feb 10;10:2213. doi: 10.1038/s41598-020-59009-z (PMC7010756; doi:10.1038/s41598-020-59009-z)
Supplement: Supplementary file 1 — Supplementary Figures. [file 41598_2020_59009_MOESM1_ESM.pdf]

Pre-operative sera interleukin-6 in the diagnosis of high-grade serous ovarian cancer

Nirmala Chandralega Kampan[1,2,3], Mutsa Tatenda Madondo[1], John Reynolds[4], Julene Hallo[2], Orla M. McNally[2,5], Thomas W Jobling[6], Andrew N. Stephens[7,8], Michael A. Quinn[7], *Magdalena Plebanski[1, 9].

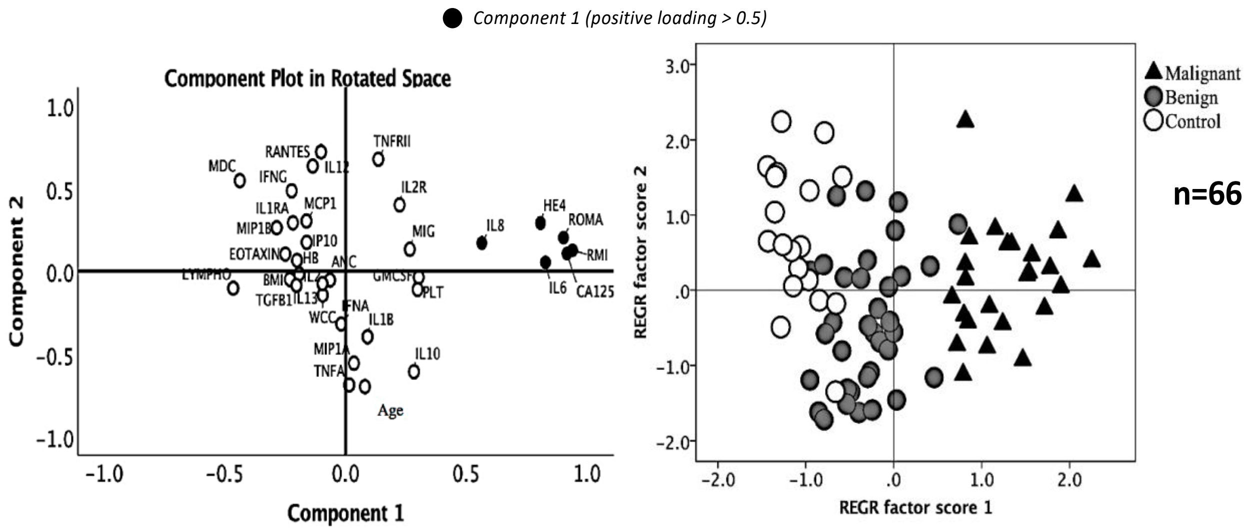

Supplementary Figure 1. Principal Component Analysis of serum soluble factor concentrations to identify factors that are predictive of malignancy. Pre-operative blood was withdrawn from 33 patients with ovarian cancer, 12 benign ovarian mass and 21 with normal ovaries. A total of 28 serum soluble factors were measured using multiplex bead immunoassay. Serum soluble factors, CA125, HE4, blood components, RMI and ROMA clinical score were included in analysis. Data reduction was conducted using principal component analysis in SPSS. The principal component revealed association between RMI, ROMA, CA125, HE4 and interleukin-6 (IL-6) and interleukin-8 (IL8) which were clustered in component 1 (black dots).

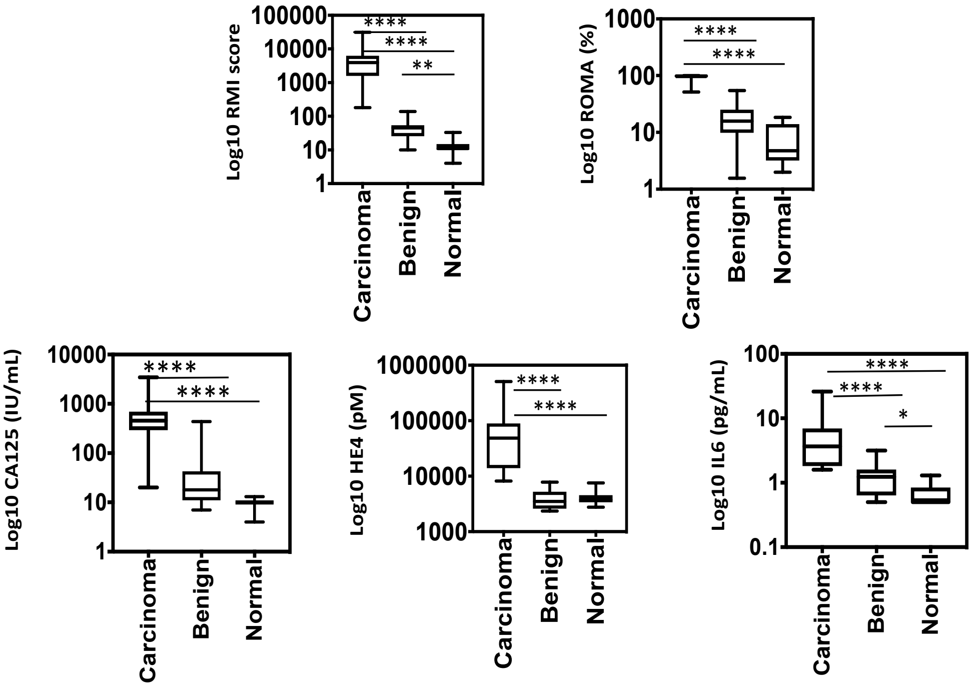

Supplementary Figure 2. Serum concentration of cytokines in advanced serous EOC patients and those with benign ovarian masses and normal ovaries in validation phase. Box-and-whisker- plots representing log10 RMI score, ROMA and serum levels of CA125, HE4 and IL-6 in patients with high-grade serous EOC, benign ovarian masses and normal ovaries. The p-value is indicated for each factor. One-way ANOVA and Dunn's multiple comparison test (post hoc); * p <0.05, ** p = 0.001-0.01, *** p = 0.0001-0.001, **** p<0.0001.

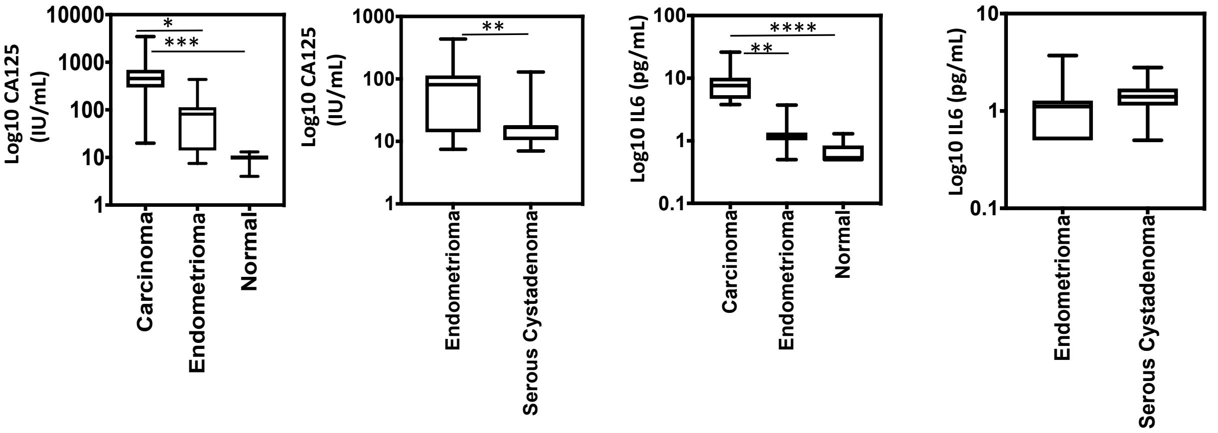

Supplementary Figure 3. Serum concentration of CA125 and IL-6 in advanced serous EOC patients and those with endometrioma and normal ovaries. Box-and-whisker- plots representing log10 serum concentrations of CA125 and IL-6 in patients with high-grade serous EOC, endometrioma and normal ovaries as well as for patients with endometrioma versus serous cystadenoma. The p-value is indicated for each factor. Kruskal-Wallis analysis followed by Dunn's multiple comparison test (post hoc) or Kolmogorov-Smirnov t-test for two small group comparison; * p <0.05, ** p = 0.001-0.01, *** p = 0.0001-0.001, **** p<0.0001.
